# Prognostic Significance of Systemic Inflammation-Based Lymphocyte- Monocyte Ratio in Patients with Lung Cancer: Based on a Large Cohort Study

**DOI:** 10.1371/journal.pone.0108062

**Published:** 2014-10-02

**Authors:** Pingping Hu, Hongchang Shen, Guanghui Wang, Ping Zhang, Qi Liu, Jiajun Du

**Affiliations:** 1 Institute of Oncology, Shandong Provincial Hospital Affiliated to Shandong University, Shandong University, Jinan, China; 2 Department of Oncology, Shandong Provincial Hospital Affiliated to Shandong University, Shandong University, Jinan, China; 3 Department of Thoracic Surgery, Shandong Provincial Hospital Affiliated to Shandong University, Shandong University, Jinan, China; Penn State Hershey Cancer Institute, United States of America

## Abstract

Increasing evidence indicates cancer-related inflammatory biomarkers show great promise for predicting the outcome of cancer patients. The lymphocyte- monocyte ratio (LMR) was demonstrated to be independent prognostic factor mainly in hematologic tumor. The aim of the present study was to investigate the prognostic value of LMR in operable lung cancer. We retrospectively enrolled a large cohort of patients with primary lung cancer who underwent complete resection at our institution from 2006 to 2011. Inflammatory biomarkers including lymphocyte count and monocyte count were collected from routinely performed preoperative blood tests and the LMR was calculated. Survival analyses were calculated for overall survival (OS) and disease-free survival (DFS). A total of 1453 patients were enrolled in the study. The LMR was significantly associated with OS and DFS in multivariate analyses of the whole cohort (HR = 1.522, 95% CI: 1.275–1.816 for OS, and HR = 1.338, 95% CI: 1.152–1.556 for DFS). Univariate subgroup analyses disclosed that the prognostic value was limited to patients with non-small-cell lung cancer (NSCLC) (HR: 1.824, 95% CI: 1.520–2.190), in contrast to patients with small cell lung cancer (HR: 1.718, 95% CI: 0.946–3.122). Multivariate analyses demonstrated that LMR was still an independent prognostic factor in NSCLC. LMR can be considered as a useful independent prognostic marker in patients with NSCLC after complete resection. This will provide a reliable and convenient biomarker to stratify high risk of death in patients with operable NSCLC.

## Introduction

Lung cancer remains the leading cause of cancer death worldwide [1]. Despite the improvement of treatment strategy, the outcome of lung cancer is still poor. Recent findings have revealed that the outcomes of cancer patients are not only determined by the characteristics of tumour, but also the patient-related factors. The interactions between tumour and host response have not been fully elucidated. Of great interest is the emerging role of inflammation, one hallmark of cancer [2], which affects many aspects of malignancy.

New paradigm in cancer treatment focuses on identifying prognostic indicators of malignancies which allows for the appropriate risk stratification of cancer patients and subsequent treatment allocation [3], [4]. It is now widely recognized that cancer-associated inflammation plays a key role in tumour progression and survival in a broad range of cancers [2]. Inflammatory biomarkers reflect the response of host to malignant tumors and hold great expectations for improving predictive ability of existing prognostic factors [5].

In recent years, many studies focused on the prognostic value of baseline haematological components reflecting systemic inflammatory response, especially the well investigated neutrophil–lymphocyte ratio (NLR) [6]–[8]. Meanwhile, tumour-associated monocyte, as another main regulator of cancer inflammation, also plays a vital role in systemic inflammatory response to tumor. A higher peripheral lymphocyte- monocyte ratio (LMR) has been suggested to be related to favorable prognosis in some hematology malignancies [9]–[11], while relevant studies in non-hematology malignancies only carried out recently. Few studies demonstrated a prognostic role for the peripheral LMR at diagnosis in limited cancers, such as soft tissue sarcomas [5], and nasopharyngeal carcinoma [12].

However, the prognostic value of the LMR in lung cancer has not been previously reported. The aim of the present study was to investigate the prognostic value of baseline LMR for operable lung cancer patients in a large-scale cohort study.

## Materials and Methods

### Patients

We conducted a retrospective study on consecutive lung cancer patients, who underwent complete resection at Shandong Provincial Hospital Affiliated to Shandong University between January 2006 and December 2011. The main inclusion criteria were histological diagnosis of primary lung cancer, complete surgical resection and without preoperative therapy. Patients were excluded if they died of postoperative complications or with positive margins. This research was approved by the Ethical Committee of Shandong Provincial Hospital affiliated to Shandong University and written informed consent was obtained by participants for their clinical records to be used in this study.

Tumor stages were based on the 7th edition of the TNM Classification [13]. The latest follow-up was carried out on December 2013. Patient's clinicopathological parameters and laboratory data were collected from the medical records. Clinicopathological parameters included age, sex, histopathology, smoke status, grade of tumour differentiation, TNM stage. The haematological variables including lymphocyte and monocyte count were obtained from blood tests routinely performed 1–3 days before operation, and the LMR was calculated by dividing the lymphocyte count by the monocyte count.

The primary endpoint of the present study was overall survival (OS), defined as the time between operation and death, loss to follow-up or the last follow-up. The secondary end point was disease-free survival (DFS), and failure was defined as confirmed recurrence.

### Statistical analysis

Statistical analyses were calculated by SPSS (version 20.0) software program (SPSS Inc., Chicago, IL, USA). Receiver operating characteristic (ROC) curve was used to define the optimal cut-off value of LMR as prognostic factors. Survival analyses were carried out by the Kaplan–Meier survival curves. The prognostic value of inflammatory biomarkers, were analyzed by Cox proportional hazard models. Multivariate Cox analyses were performed in a step-forward logistic regression approach. Correlations were compared by Spearman's-rho analyses. The distributions of inflammatory biomarkers were analyzed using Mann–Whitney U-test. A two tailed *p*-value ≤0.05 was considered significant.

## Results

### Patients' characteristics

A total of 1453 patients were enrolled in our study. All patients underwent curative surgical resection. The basic characteristics of the patients are detailed in Table 1. These patients including 1035 men and 418 women with median age of 59.0 years old (range 20–84 years). Five hundred and nine patients (35.03%) died at the end of last follow-up.

**Table 1:** Basic characteristics of enrolled patients.

	Total (1453)
Sex	
Male	1035
Female	418
Age	
≤65	1058
>65	395
TNM Stage	
I	603
II	377
III	473
Smoke status	
Ex-smoker	130
Current smoker	782
Non-smoker	541
Histology	
NSCLC	1356
Small-cell lung cancer	97
Differentiation	
Grade 1–2	1117
Grade 3–4	336

Abbreviations: TNM, tumour node metastasis; NSCLC, non-small-cell lung cancer.

### Survival analyses

As for the lymphocyte, the median value was 1.75×10^9^/L (range 0.30–6.95×10^9^/L). For monocyte, the median value was 0.46×10^9^/L (range 0.01–1.67×10^9^/L). The cut-off values determined by ROC curves for lymphocyte, monocyte, and LMR were 1.705, 0.485 and 3.68, respectively.

Based on the cut-off value, we separated the patients into two groups (low value group and high value group). Survival analyses in relation to inflammatory biomarkers and patients' outcome were performed. In the univariate analysis about OS, lymphocyte, monocyte were both proved to be significant factors, with hazard ratio (HR)  = 1.26 (95% confidence interval [CI]: 1.053–1.508, low: high) for lymphocyte and HR = 0.684 (95% CI 0.575–0.815, low: high) for monocyte. To identify the optimal inflammatory biomarker for patients' outcome, we evaluated the prognostic value of LMR. The HR for LMR was 1.829 (95% CI 1.536–2.178, low: high), implying more important prognostic value. In the analysis about DFS, the HR for LMR was 1.541 (95% CI 1.329–1.787). Other identified prognostic factors for OS and DFS including sex, age, smoke status, and TNM stage.

To determine the independent prognostic factors for OS and DFS, multivariate analyses using Cox proportional hazard models including above variates were performed. We identified that age, TNM stage and LMR were significant prognostic factors for OS and DFS (Table 2). The HRs of LMR were 1.510 (95% CI 1.265–1.803) for OS and 1.541 (95% CI 1.329–1.787) for DFS, respectively.

**Table 2:** Survival analyses of inflammatory biomarkers and clinicopatholigic factors.

	OS				DFS			
	Univariate analysis		Multivariate analysis		Univariate analysis		Multivariate analysis	
Variable	HR (95% CI)	*p*	HR (95% CI)	*p*	HR (95% CI)	*p*	HR (95% CI)	*p*
Sex								
Male	1.404 (1.144–1.723)	0.001	*		1.246 (1.053–1.474)	0.009	*	
Female	1				1			
Age								
≤65	0.711 (0.591–0.855)	<0.001	0.554 (0.459–0.669)	<0.001	0.824 (0.701–0.968)	0.019	0.695 (0.590–0.819)	< 0.001
>65	1		1		1			
Smoke status								
Ex-smokers	1.614 (1.183–2.204)	0.003	*		1.576 (1.218–2.040)	0.001	*	
Current smokers	1.415 (1.167–1.716)	<0.001			1.225 (1.043–1.439)	0.013		
Never smokers	1							
TNM stage								
I	0.216 (0.171–0.273)	<0.001	0.206 (0.162–0.261)	<0.001	0.346 (0.289–0.414)	<0.001	0.340 (0.283–0.409)	<0.001
II	0.611 (0.500–0.748)	<0.001	0.562 (0.458–0.688)	<0.001	0.668 (0.559–0.800)	<0.001	0.635 (0.530–0.760)	<0.001
III	1		1		1			
LMR								
Low	1.829 (1.536–2.178)	<0.001	1.510 (1.265–1.803)	<0.001	1.541 (1.329–1.787)	<0.001	1.330 (1.144–1.546)	<0.001
High	1		1		1		1	

Abbreviations: HR, hazard ratio; CI, confidence interval; TNM, tumour node metastasis; OS, overall survival; DFS, disease-free survival.

*, Not in the final step of multivariate analysis.

Further analyses were performed in subgroups of small cell lung cancer and non-small-cell lung cancer (NSCLC). In univariate analysis, the prognostic value was limited to patients with NSCLC (HR: 1.824, 95% CI: 1.520–2.190), in contrast to patients with small cell lung cancer (HR: 1.718, 95% CI: 0.946–3.122). Multivariate analyses found LMR was still an independent prognostic factor in NSCLC (HR = 1.511, 95% CI: 1.256–1.819 for OS; and HR = 1.335, 95% CI: 1.141–1.561 for DFS).

**Figure 1:**
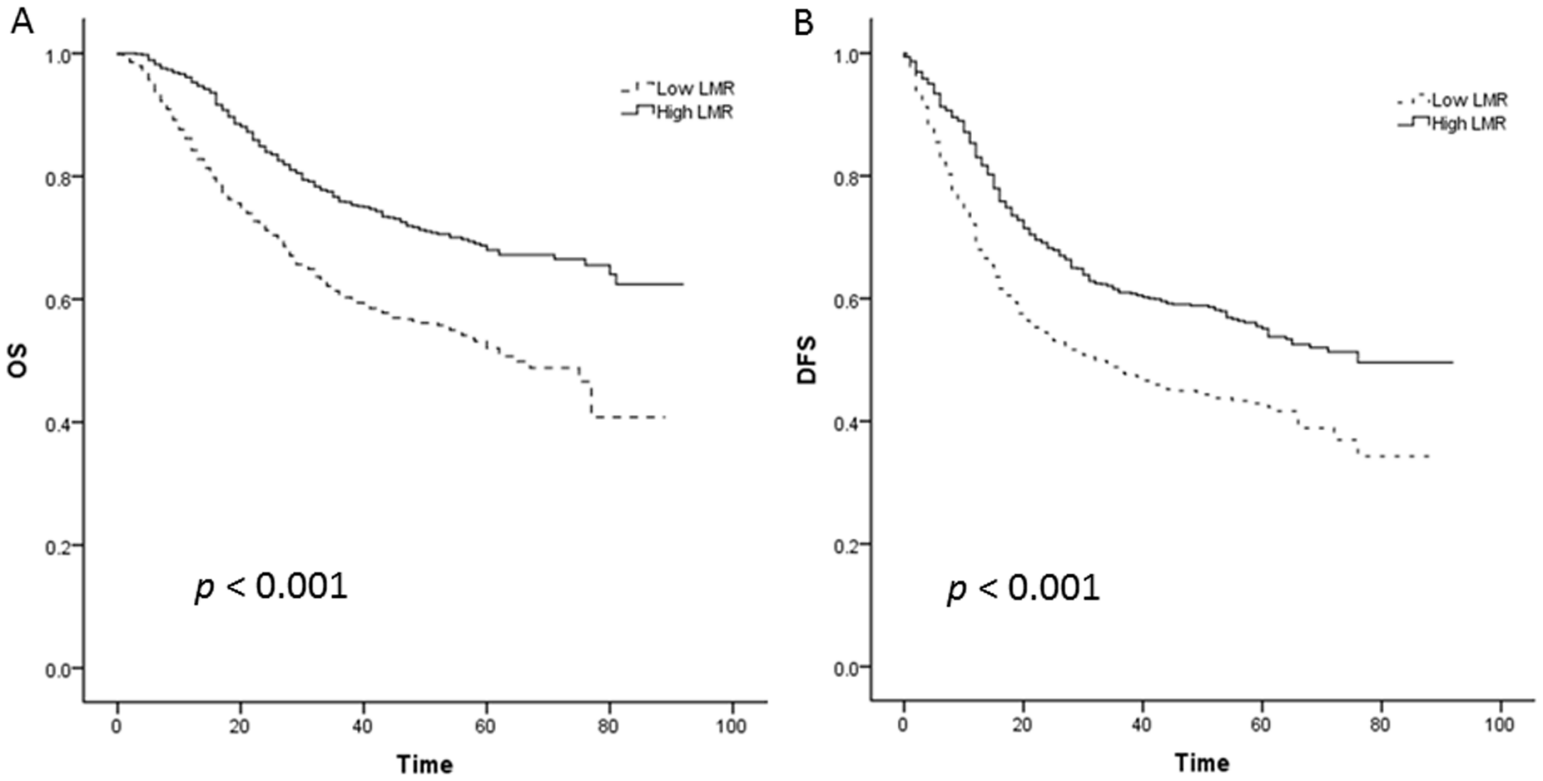
Kaplan–Meier curves for overall lung cancer patients. A: Kaplan–Meier curve of overall survival (OS) for overall lung cancer patients; B: Kaplan–Meier curve of disease-free survival (DFS) for overall lung cancer patients.

**Figure 2:**
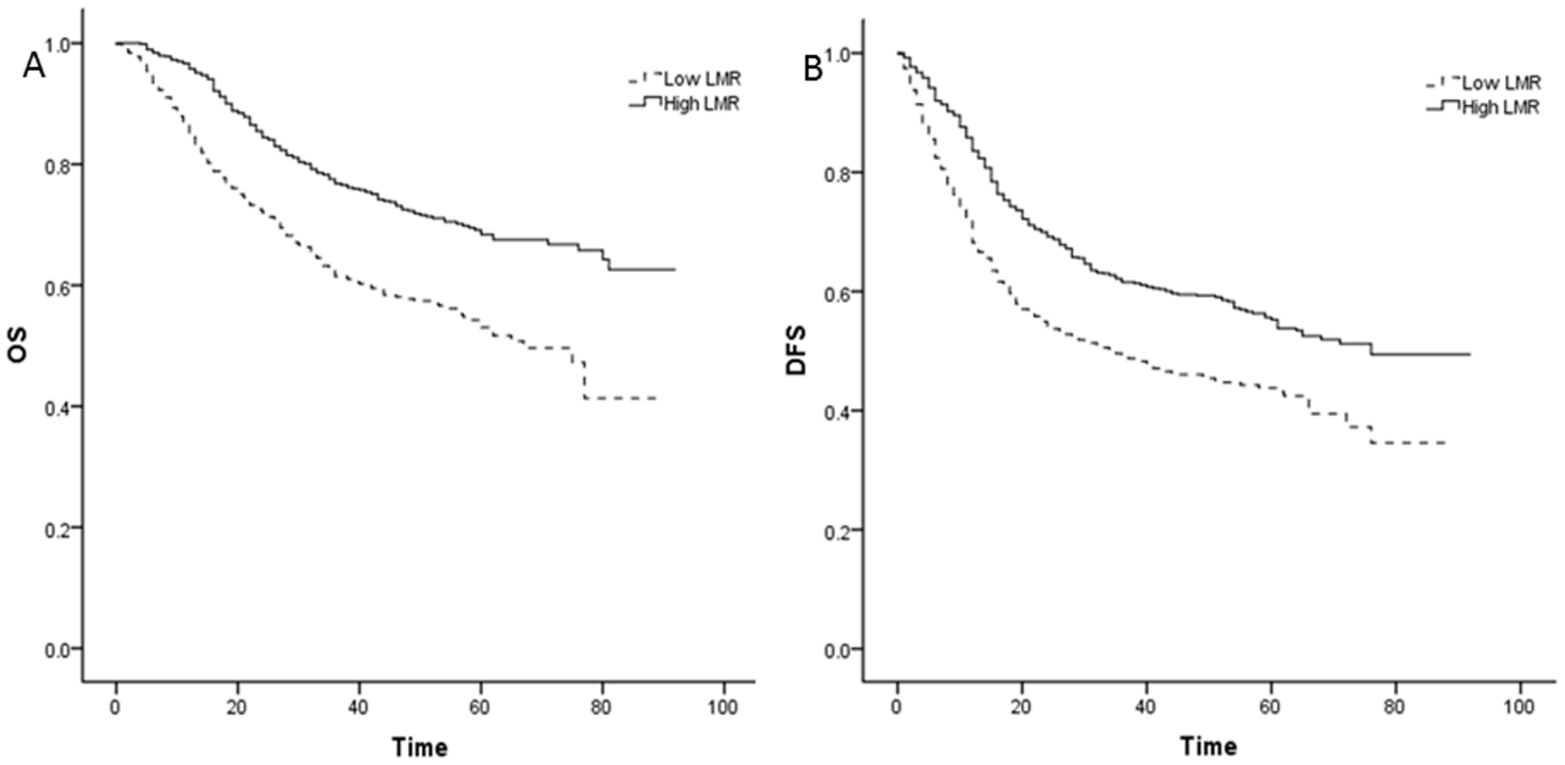
Kaplan–Meier curves for non-small-cell lung cancer (NSCLC). A: Kaplan–Meier curve of OS for NSCLC; B: Kaplan–Meier curve of DFS for NSCLC.

Kaplan–Meier curves revealed that a low LMR was significantly associated with decreased OS and DFS for overall lung cancer patients (Figure 1A, B) and NSCLC patients (Figure 2A, B), with all *p*<0.001 by log- rank test.

### Correlations between LMR and clinicopathological factors

The correlations between the LMR and clinicopathological factors are shown in Table 3. In Spearman's-rho analyses, there was no significant correlation for age (*p* = 0.064). Though there were statistically significant correlation between LMR and factors including smoke status, TNM stage, T stage, N stage, and differentiation, the correlation coefficient were low (correlation coefficient  = 0.218, −0.161, −.0221, −0.081 and −0.160, respectively. Table 3).

**Table 3:** Correlations between LMR and clinicopathological factors.

Clinicopathological factors	Correlation coefficient	*p* value
Age	−0.049	0.064
Smoke status	0.218	<0.001
TNM Stage	−0.161	<0.001
T stage	−0.221	<0.001
N stage	−0.081	0.002
Differentiation	−0.160	<0.001

Abbreviations: LMR, lymphocyte- monocyte ratio; TNM, tumour node metastasis.

## Discussion

In the present study, we carried out a large-scale retrospective cohort study, and demonstrated that high LMR was a favorable prognostic factor for OS and DFS in lung cancer. In sub-analyses, the relationship was limited to NSCLC instead of small cell lung cancer. But the result of small cell lung cancer should be interpreted cautiously, because only limited patients were enrolled in the present study. Previous studies identified potentially prognostic cell ratios including NLR [8], [14], [15], platelets/lymphocyte ratio (PLR) [14], and LMR [5], [10], [11], [16]. Furthermore, the prognostic value of LMR has been investigated mainly in hematological cancer, while the data in solid tumor are sparse. Despite of the published studies, data of LMR on lung cancer patients have not been reported. This is the first study investigating the prognostic value of LMR in lung cancer to the best of our knowledge. The findings are in line with former published studies from hematological and some solid cancers [9], [10], [12], [16]–[18], and it will represent another reliable and convenient biomarker reflecting the systemic inflammatory response in the tumour microenvironment.

There is substantial evidence in tumour that the systemic immune response of host is a vital independent prognostic factor. Inflammation in microenvironment promotes tumorigenesis, progression and metastasis [19]. The knowledge of the bilateral influence of inflammation and cancer was described over a century ago, by Rudolf Virchow [20]. Solid tumors are generally infiltrated with leukocyte subsets, among which monocytes and lymphocytes play major roles in the inflammatory response. Either each of these two leukocyte subsets or combination of peripheral LMR, has been demonstrated independently associated with the prognosis of various cancers. A lower lymphocyte count and high monocyte count were both significantly related to mortality in ovarian cancer [21]. High number of circulating monocytes was associated with poor progression-free survival and OS in bevacizumab-treated non-small-cell lung cancer (NSCLC) patients [22]. Increase in perioperative monocyte less than twice was an independent risk factor for OS after hepatic resection in patients with colorectal liver metastasis [23]. A higher baseline monocyte count was an independent predictor of poor outcome with locally advanced cervical squamous cell carcinoma [24]. The high preoperative LMR was significantly associated with long time to recurrence and OS in patients with stage III colon cancer [25].

Although the mechanisms for the relationship between increasing LMR and dismal outcome have not been elucidated, a high monocyte count or a low lymphocyte count has been reported as an adverse biomarker of prognosis in various cancers. Extensive studies now indicate that monocytes generally infiltrate in most solid malignancies. Tumor-associated macrophages (TAMs), deriving from the monocytic precursors circulating in blood [26], are recruited at the tumor via chemotaxis and constitute a significant component of the inflammatory infiltrate of several malignances [26], [27]. The tumor-promoting TAMs orchestrate tumor microenvironment by several mechanisms including regulating senescence, interacting with and contributing to extracellular matrix remodeling, promoting proliferation, progression, angiogenesis and lymphangiogenesis [28], [29]. The circulating monocytes in blood may reflect the formation or presence of TAMs [5]. Therefore, it is well recognized as a useful biomarker for high tumour burden in cancer.

Contrary to pro-tumour activity of TAM, lymphocytes play crucial roles in host cell-mediated immunity regulation which is important to destruct residual malignant cells and related micrometastases [30]. A low circulating lymphocyte count is recognized as a predictor of adverse outcome in patients with different cancers. Locally advanced rectal cancer patients with high lymphocyte ratio before neoadjuvant chemoradiotherapy had better tumor response and favourable 5-year DFS and OS [31]. Preoperative lymphocyte count was an independent favorable prognostic biomarker of DFS in NSCLC [32]. In another study, preoperative lymphocyte count, was proved to be correlated to vascular invasion, and was an independent prognostic marker in node-negative NSCLC [33].

Although circulating monocyte or lymphocyte count alone could work as a prognostic factor, the LMR was demonstrated to outperform better than either of them. As lymphocyte count was recognized as a predictor of favorable outcome while monocyte count was a predictor of poor outcome. The combined LMR derived from above variables expanded the favorable effect against the unfavorable effect, therefore, the prognostic value was enlarged. Meanwhile, the interaction between these two leukocyte subsets might be contributing into the performance [12].

Besides the well-recognized clinicopathological prognostic markers, recent studies have focused on identifying new markers, which can be highly reproducible, easily obtainable, inexpensive and reliable. Haematological tests are one of the routine tests carried out in cancer patients, and the LMR represents a reliable and convenient biomarker reflecting the degree of the systemic inflammatory response in NSCLC. LMR may also be useful in directing therapeutic decision-making such as directing adjuvant therapy for high-risk patients. Meanwhile, it is possible that while prognostic, this measurement may not allow exclusion or inclusion of patients for such adjuvant therapy. This would need to be determined in future prospective clinical trials.

## Conclusion

In conclusion, we firstly demonstrated that pretreatment circulating LMR can be considered as a useful independent prognostic marker in patients with NSCLC after complete resection. This will provide a reliable and convenient biomarker to stratify high risk of death in patients with operable NSCLC.
